# Longitudinal Trajectory of Free Fatty Acids in Pregnancy According to First-Trimester Maternal Metabolic Status and the Presence of Gestational Diabetes

**DOI:** 10.3390/metabo15050320

**Published:** 2025-05-11

**Authors:** Otilia Perichart-Perera, Isabel González-Ludlow, Omar Piña-Ramírez, Maricruz Tolentino-Dolores, Guadalupe Estrada-Gutierrez, Sandra B. Parra-Hernández, Maribel Sánchez-Martínez, Omar Granados-Portillo, Ameyalli M. Rodríguez-Cano

**Affiliations:** 1 Nutrition and Bioprogramming Coordination, National Institute of Perinatology, Mexico City 11000, Mexico; otiliaperichart@inper.gob.mx (O.P.-P.);; 2 Bioinformatic and Statistical Analysis Department, National Institute of Perinatology, Mexico City 11000, Mexico; omar.pina@inper.gob.mx; 3 Department of Immunobiochemistry, National Institute of Perinatology, Mexico City 11000, Mexico; gpestrad@gmail.com (G.E.-G.); sandrabpahdz@gmail.com (S.B.P.-H.); maribel1971sm@gmail.com (M.S.-M.); 4 Nutrition Physiology Department, National Institute of Nutrition and Health Sciences, Mexico City 14080, Mexico; omargranadosp@incmnsz.mx

**Keywords:** obesity, lipids, insulin resistance, gestational diabetes

## Abstract

Background/Objectives: Maternal free fatty acids (FFAs) play a critical role in maternal metabolism, fetal growth, and pregnancy outcomes. However, their relationship with maternal metabolic status in early pregnancy and the subsequent development of gestational diabetes mellitus (GDM) remains unclear. Aim: Assess the trajectory of FFA concentrations during pregnancy, considering first-trimester metabolic status (obesity, insulin resistance—IR) and the development of GDM, and evaluate whether first-trimester FFA is a relevant risk factor for GDM. Methods: A case–control study nested within the OBESO cohort (Mexico City, pregnant women and their children), classified women according to first-trimester metabolic status (pregestational body mass index—pBMI, insulin resistance homeostasis model assessment—HOMA-IR > 1.6), as well as the presence of GDM: Group 1 (normal weight without IR, n = 60), Group 2 (obesity without IR, no GDM, n = 20), Group 3 (obesity with IR, no GDM, n = 20), and Group 4 (obesity with IR, with GDM, n = 9). FFA concentrations were measured each trimester. Statistical analyses included repeated measures ANOVA and logistic regression models. Results: FFA concentrations were the highest in Group 4 across all trimesters (*p* < 0.05). FFAs decreased throughout pregnancy in all groups (*p* = 0.023), with the most significant decline from the first to the third trimester (*p* < 0.001). The greatest reduction occurred in Group 4 (*p* < 0.001), followed by Group 3. Multivariate logistic regression showed no association between first-trimester FFAs and the development of GDM. Higher gestational weight gain was associated with a higher GDM risk (OR: 1.22, 95%CI: 1.01–1.48), when the FFAs difference was accounted for. Conclusions: FFA levels are higher in women with GDM compared with women with obesity or a normal weight. However, FFAs progressively decline from the first to the third trimester, with the most pronounced decrease in women with obesity, IR, and GDM.

## 1. Introduction

Obesity is a major global public health concern, disproportionately affecting women. Over the past three decades, its prevalence in females has increased by 104% and projections estimate that by 2050, 60% of women worldwide will be overweight or will have obesity [1]. In Mexico, 40% of adult women already live with obesity [2]. In women of reproductive age, pregestational obesity poses clinical challenges, including an increased risk of gestational diabetes mellitus (GDM) and other adverse perinatal outcomes. Additionally, it influences fetal metabolic programming, with long-term health implications for the offspring [3,4].

Recently, the obesity discussion has shifted toward the better characterization of the disease to improve diagnosis and treatment [5,6]. In general, there exists the “metabolically healthy” obesity type, which presents as high body mass index (BMI) or adiposity (in non-central regions) without apparent metabolic alterations, and the “metabolically unhealthy” type, which is associated with higher visceral fat and different metabolic alterations (insulin resistance, dyslipidemia, high oxidative stress, and inflammation) [5]. In pregnancy, obesity classification has been centered on pregestational BMI (pBMI), often based on self-reported pregestational weight. Women with pregestational obesity may or may not have metabolic disturbances, though they frequently present elevated circulating insulin, lipids, and leptin levels, and an increased risk of developing GDM [7,8,9].

Free fatty acids (FFAs) are individual fatty acid molecules that are not esterified to glycerol; they are released during the hydrolysis of triglycerides, a reaction catalyzed by the enzyme, lipoprotein lipase. They are essential for energy production, cell signaling, and various other physiological processes. Circulating FFAs have gained attention due to their role in promoting insulin resistance and altering insulin secretion. Studies have shown increased FFA levels in adults with obesity or type 2 diabetes mellitus [10]. Excessive adiposity results in higher lipolysis, leading to an increase in plasma FFAs, which inhibits insulin-mediated glucose uptake. Chronic elevation of FFAs may compromise β-cell compensatory mechanisms [10], promoting insulin resistance. Lipid metabolism alterations are more frequent in obesity and diabetes-complicated pregnancies, including higher lipolysis [11,12]. Notably, higher fatty acid concentrations in early pregnancy have been linked to an increased risk of developing GDM later in gestation [13].

The longitudinal behavior of FFAs during pregnancy has been scarcely studied, with a high variability between specific fatty acids. Higher concentrations of FFAs have been reported pre-pregnancy or early pregnancy compared with late pregnancy [11,14,15]. A meta-analysis of cross-sectional studies reported that women with GDM had significantly higher FFA levels than healthy pregnant controls, suggesting that elevated FFAs may be a potential marker of insulin resistance and a possible predictor of GDM [14]. In a case–control study, the change in specific FFA concentrations was associated with a higher risk of GDM, and a higher total FFAs score also increased this risk [16]. On the other hand, increased rates of lipolysis in late pregnancy have also been correlated with fetal fat accretion [11] and elevated postprandial FFAs in women with GDM have been associated with a higher risk of delivering large for gestational age newborns [17]. However, FFA dynamics are complex and may vary depending on maternal metabolic risk profiles in early pregnancy.

The early and accurate identification of women at higher metabolic risk and, consequently, at higher risk of developing GDM could facilitate timely preventive interventions and individualized monitoring strategies. This study aimed to assess the trajectory of FFAs during pregnancy, considering maternal obesity and insulin resistance in the first trimester and the development of GDM. In addition, we investigated whether FFA in the first trimester, alongside other metabolic variables, are relevant risk factors of GDM.

## 2. Materials and Methods

### 2.1. Study Design and Population

This study is a case–control study nested in the OBESO cohort (Origen Bioquímico y Epigenético del Sobrepeso y la Obesidad), which is being conducted at the Instituto Nacional de Perinatología Isidro Espinosa de los Reyes, Mexico City, Mexico. The study was approved by the Institutional Ethics and Research Committees (No. 3300–11402-01–575-17, No. 2024-1-14), and all procedures were conducted according to the Declaration of Helsinki. Women were recruited in the first trimester (11.3–13.6 weeks) of pregnancy based on the OBESO’s cohort inclusion and exclusion criteria. All women were screened in consecutive order and selected by convenience according to the following inclusion criteria: adult pregnant women with a pregestational body mass index (preBMI) ≥ 18.5, without previous diseases (type 2 diabetes mellitus, hypertension, uncontrolled thyroid disorders, or heart, renal, hepatic or autoimmune diseases). Women with fetuses with congenital structural malformations, pregnancy loss, or who did not complete their follow-up visit were excluded [18]. During the first-trimester visit, sociodemographic and clinical information was collected, including age (years), education (categorized as middle-low for middle and high school/technical education and High for university degree), and parity (nulliparity—no previous childbirth and multiparity—≥1 childbirth). To compute p-BMI, women self-reported their pregestational weight. Height was measured to the nearest 0.1 cm using a digital fixed stadiometer (model 264, SECA, Hamburg, Germany) (Lohman’s technique) [19]. Normal weight was defined as a pBMI > 18.5 and <25.0, and obesity was defined as a pBMI ≥ 30 [20]. Current weight was measured to estimate gestational weight gain (GWG) in the first trimester.

### 2.2. Metabolic Assessment

A fasting (minimum 8 h) blood sample was collected in vacutainer tubes (Becton-Dickinson, Franklin Lakes, NJ, USA) and centrifugated for 10 min at 1900× *g* in the Research Unit at our hospital. Serum samples were stored at −70 °C until analysis. Glucose levels were measured using the final point colorimetric enzymatic method with an automated biochemistry analyzer (Respons 910, Diasys, Halzeim, Germany). Insulin was measured on an ARCHITECT i1000SR Clinical Chemistry Analyzer (Abbot Diagnostics, Abbott Park, IL, USA). The sensitivity for glucose measurement was 1 mg/dL and for insulin 1 μU/L, with a coefficient of variation less than 4% and <7%, respectively. The homeostatic model assessment for insulin resistance (HOMA-IR = [fasting serum insulin (μU/mL) × fasting serum glucose (mg/dL)]/405) was used to evaluate insulin resistance (IR), which was defined as a value > 1.6. A 75 g, 2 h oral glucose tolerance test was performed at 24–28 weeks of gestation for GDM diagnosis. The International Association of Diabetes and Pregnancy Study Groups (IADPSG) criteria were used (≥1 abnormal glucose values: fasting ≥ 92 mg/dL, one h ≥ 180 mg/dL, two h ≥ 153 mg/dL) [21]. Women diagnosed with GDM (Group 4) were referred to the usual treatment for diabetes in pregnancy as stated in our institutional guidelines, including medical nutrition therapy and pharmacologic treatment (metformin or insulin) if glycemic goals were not met (fasting or preprandial glucose > 95 mg/dL, one-hour postprandial glucose > 140 mg/dL).

### 2.3. Study Groups

For this study, four groups were selected according to metabolic status in the first trimester and the development of GDM as follows:

- Group 1 (normal weight): Normal-weight women without insulin resistance at first trimester, without GDM.
- Group 2 (obesity): Women with obesity without insulin resistance at first trimester, without GDM.
- Group 3 (obesity and IR): Women with obesity with insulin resistance at first trimester, without GDM.
- Group 4 (obesity, IR, and GDM): Women with obesity and insulin resistance at first trimester, with GDM.

### 2.4. Free Fatty Acids and Lipid Measuerments

FFAs were measured using the final point colorimetric enzymatic method (Trinder) with an automated biochemistry analyzer (Respons 910, Diasys, Halzeim, Germany), using coenzyme A, acyl-CoA-synthetase, acyl-CoA-oxidase, MgCl_2_, and peroxidase as reagents. To consider a broader metabolic profile, we also measured triglycerides (TG), and high-density lipoproteins cholesterol (HDL-C) concentrations with the same methodology. An additional fasting blood sample was collected to complete the longitudinal FFA measurements (second trimester: 18.0–24.0 weeks, third trimester: 28.0–34.6 weeks). The intra-assay coefficient of variation was <5%, with a sensitivity of 0.007 mmol/L. For TG and HDL-chol, coefficient of variation was <4%, with sensitivities of 3 mg/dL and 5 mg/dL, respectively. The difference in FFAs was calculated by subtracting third-trimester from first-trimester values.

### 2.5. Statystical Analysis

All statistical analyses were conducted using Python (v3.9) with the pingouin and stats models packages. Descriptive analyses of metabolic variables during the first trimester were performed using one-way ANOVA or the Kruskal–Wallis test, depending on the Shapiro–Wilk and Levene’s tests for normality and homoscedasticity assumptions, respectively. Post hoc comparisons were adjusted with Bonferroni correction.

A repeated measures ANOVA with a mixed-effects design was performed to evaluate FFAs across trimesters, considering trimester as the within-subject factor and group as the between-subject factor. Mauchly’s test was conducted to assess sphericity, and the Greenhouse–Geisser correction was utilized. Post hoc comparisons were performed using a *t*-test for the group dimension and a Wilcoxon signed-rank test for the trimester dimension; the Bonferroni correction was applied. A linear mixed-effects model (LMM) was estimated to evaluate the influence of GWG as a covariate in the longitudinal model.

Multiple logistic regression models were implemented to explore the association between first-trimester metabolic markers and GDM. All models included p-BMI, GWG, HOMA-IR, and TG. The first model included variables as mentioned; the second model included glucose and insulin instead of HOMA-IR; and the third model included the difference in FFAs (from third to first trimester) instead of FFA concentrations.

Additional repeated measures ANOVAs were conducted to examine the interactions between FFAs, maternal age (>35 years old), and educational level (dichotomized as low and high level). These models were specified analogously to the primary repeated measures ANOVA. All significance thresholds were set at *p* < 0.05. Bayes Factor (BF10) for parametric tests and Hedges’ g for both parametric and non-parametric tests, as metrics of effect size, were computed for all pairwise comparisons. Robust standard errors (HC0) were used in the logistic regression models.

## 3. Results

From the total of 483 women participating in the OBESO cohort between 2017 and 2023, 108 were included in this study based on their weight and metabolic status. Women were excluded if they lacked an OGTT at 24–28 weeks (n = 217), had incomplete baseline metabolic marker measurements (n = 115), or were lost during follow-up (n = 43). The eligible participants were assigned to one of the four study groups: Group 1: n = 60, Group 2: n = 20, Group 3: n = 19, and Group 4: n = 9.

The mean age of the women was 29.0 ± 5.29 years (19–41 years). Most were married or cohabiting (77.7%%, n = 84). Low-middle education was the more frequent level of study (66.4% of women), while only 33% of women had undertaken higher education. Multiparity was reported in 38.9% (n = 42) of participants. No differences were observed in these variables by study group. Among the women who developed GDM (group 4), three out of nine initiated metformin treatment after diagnosis, while none required insulin.

Table 1 describes the women’s first-trimester weight and metabolic status according to the study group. Post hoc analyses revealed higher HOMA-IR values in women in Group 4 and Group 3, compared with Group 2 and 1 (*p* ≤ 0.002), and higher p-BMI in Group 4, 3, and 2 compared with women in Group 1 (*p* < 0.001), according to the study inclusion criteria. Insulin levels were significantly higher in women in Groups 3 and 4 compared with women in Groups 2 and 1 (*p* < 0.001), while mean glucose was higher in Group 3, compared with Group 1 (*p* < 0.001). GWG in this period was highly variable, where Group 2 showed lower GWG than Group 1 (*p* = 0.006) and Group 4 (*p* = 0.02). The lowest TG concentrations were found in Group 1, which were significantly lower than Group 2 (*p* = 0.02). HDL-C was not different between groups.

FFA concentrations by trimester were higher in the women in Group 4 (obesity, IR, and GDM) compared with the women in Group 1 (normal weight) (Table 2). Mean FFA differences were not different between groups (*p* = 0.09). Within group 4, third-trimester FFA concentrations were not different among the women who used metformin (0.403 ± 0.08 mmol/L, n = 3) compared with those who did not (0.476 ± 0.08 mmol/L, n = 6).

Overall, FFA concentrations significantly decreased throughout pregnancy in all women (*p* = 0.023). Post hoc analysis by trimester showed the most pronounced decline from the first to the third trimester (BF10: >>10,000; hedges: 0.77, *p* < 0.001), with a smaller, but significant reduction from the first to the second trimester (BF10: 761.61, hedges: 0.46).

**Figure 1:**
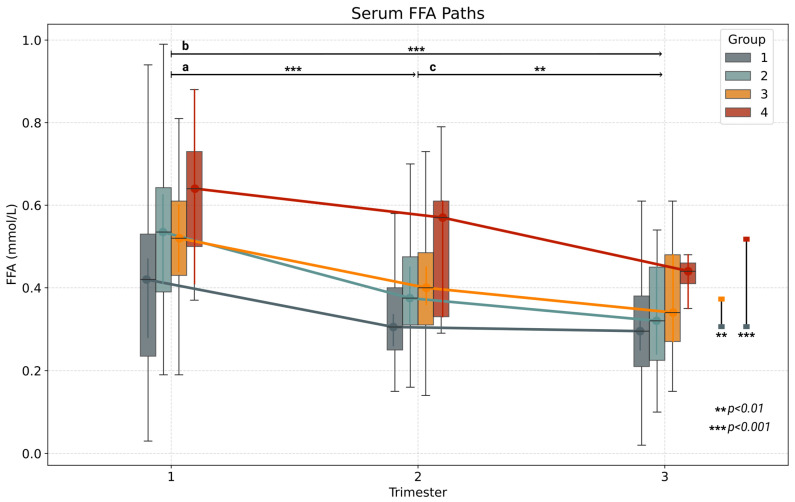
FFA concentrations throughout pregnancy in women by study group. FFAs = free fatty acids. Group 1 = normal-weight women. Group 2 = women with obesity (without insulin resistance, without GDM). Group 3 = women with obesity with insulin resistance (without GDM). Group 4 = women with obesity with insulin resistance (with GDM). Horizontal lines represent differences between trimesters. ^a^. Changes from first to second trimester. ^b^. Changes from first to third trimester. ^c^. Changes from first to third trimester. Vertical lines show differences between groups across time. ** *p* < 0.01, *** *p <* 0.0001.

When analyzed by study group, FFA concentrations differed significantly between normal-weight women (Group 1) and women in Group 3 and Group 4. The most pronounced FFA decrease occurred in women with obesity, IR, and GDM (Group 4) when compared with normal-weight women (Group 1) (BF10: 4750.19, hedges: 1.09, *p* < 0.001) (Figure 1). A significant but less marked decline was also observed in Group 3 (BF10: 49.19, hedges: 0.53, *p* = 0.003). The model did not vary significantly when first-trimester GWG was considered.

A logistic regression model including all women, irrespective of the study group assigned, did not show a significant association between first-trimester maternal metabolic markers (p-BMI, HOMA-IR, FFA, TG, and GWG) and the development of GDM (Table 3). The results did not change when first-trimester glucose and insulin were substituted for HOMA-IR in the model. When the model incorporated the difference in FFAs between the third and first trimester, higher GWG was significantly associated with an increased risk of GDM (OR: 1.22, 95% CI: 1.01–1.48) (Table 3).

## 4. Discussion

To our knowledge, this is the first study that longitudinally describes maternal FFA concentrations throughout pregnancy while considering both early metabolic status (weight status and insulin resistance) and the development of GDM.

Our findings align with previous reports indicating that pregnant women with GDM have higher FFA concentrations than healthy women [22,23]. However, most existing evidence derived from cross-sectional case–control studies, which report FFA concentrations mainly in the second or third trimester of pregnancy, often without considering maternal weight or metabolic status. A meta-analysis of 12 studies (2019) and a more recent narrative review reported higher FFA levels in women with GDM (SMD: 0.86; 0.54–1.18), with the highest values observed in the second trimester [14].

By analyzing FFA trajectories throughout pregnancy, we observed that, overall, FFA levels were highest in the first trimester and progressively decreased in the second and third trimesters, with a more pronounced decline in women with obesity, IR, and GDM and women with obesity and IR (no GDM), compared with normal-weight women. A recent longitudinal study (CLIMB cohort) examining individual FFAs trajectories (saturated, monounsaturated, and polyunsaturated) throughout pregnancy according to the presence of GDM, observed highly variable patterns, with some FFAs significantly increasing or decreasing in women with GDM [23]. The specific decline in FFAs in women with obesity and IR, irrespective of GDM development, observed in our study, suggests that early maternal metabolic status influences FFAs regulation throughout pregnancy, which may have implications for fetal growth. This reduction may reflect compensatory mechanisms for the already higher concentrations in women with obesity and IR. One pathway includes increased placental FFAs transport to the fetus, contributing to fetal fat accretion. In pregnant women, a metabolic shift toward lipogenesis and increasing fat depots is consistent with higher insulin and FFA levels. In women with GDM, a significant upregulation of fatty acid translocase (CD36) and fatty acid binding proteins (FABP1, FABP4, and FABP5), along with a decreased expression of endothelial lipase and fatty acid transport (FATP4), has been reported [24]. A case–control study within the PREOBE cohort, demonstrated that a higher pBMI or the presence of GDM alters gene expression involved in fatty acid uptake and metabolism, as well as placental fatty acid composition [25]. In addition, a longitudinal study of 1156 mother–child pairs found that GDM was associated with an increased risk of childhood overweight/obesity, central obesity, and high body fat (OR 1.41–1.57 at 5.9 years of age and 1.73–2.03 at 8.3 years of age) compared with children of mothers without GDM [26]. However, to our knowledge, no studies have specifically evaluated changes in fat mass in women with GDM.

The observation that FFA concentrations were higher in the first trimester and decreased thereafter had been scarcely reported in longitudinal studies. A small study on lipid and carbohydrate metabolism described higher pre-pregnancy FFA concentrations that significantly decrease during pregnancy [11]. Similarly, another retrospective cohort study in women with different subgroups of hypertensive disorders of pregnancy reported higher FFA concentrations at 4–16 weeks compared with 28–42 weeks, with a mean difference of −0.12 ± 0.30 mmol/L, which is similar to the difference we observed in normal-weight women [15].

Traditionally, obesity and metabolic risk during pregnancy have relied on the pBMI and GDM diagnosis, which is recommended to be performed completed at 24–28 weeks of gestation [21,27]. This timing may be too late for effective and timely interventions. Recent initiatives suggest early screening for mild or early GDM in the first trimester, using markers such as HOMA-IR or fasting glucose [28]. However, these approaches still fail to comprehensively assess metabolic risk in women who start pregnancy with obesity. In line with this, novel subtypes of metabolic risk (high HDL-C, dyslipidemic–high TG, dyslipidemic–high FFA, and insulin resistant–hyperglycemic) in pregnancy have been proposed and studied, showing differences in infant fat mass between these groups [29]. We were particularly interested in the first trimester FFA concentrations and other metabolic risk markers (obesity, HOMA-IR, TG, HDL-C, and GWG). FFAs were higher in women with obesity, IR, and GDM. Notably, the FFA trajectories were different in healthy, normal-weight women and metabolically healthy women with obesity compared with those who had obesity and IR, regardless of whether they developed GDM. First-trimester GWG was also different between groups, likely reflecting that early excessive accumulation of adipose tissue may contribute to the development of IR or GDM. The Generation R study reported that higher GWG during pregnancy was associated with increased mid-pregnancy fatty acid levels, independent of p-BMI [30]. Furthermore, early maternal body composition components have been associated with GDM risk, independent of p-BMI or GWG [31]. Interestingly, when analyzing all women included (regardless of study groups), we did not find an association between first-trimester metabolic markers and the development of GDM, which may be partially explained by the limited study design (case–control) and the small sample size in the GDM group (n = 9). However, we observed a modestly increased risk of GDM in women with higher GWG in the first trimester (Table 3). In a prospective study in Chinese women, an excessive GWG in the first trimester (>2.0 kg, according to IOM reported usual gain) increased the risk of GDM across all maternal weight categories (underweight, normal, overweight, and obesity) after adjusting by multiple confounders [32]. Moreover, our findings indicate that normal-weight women and those with metabolically healthy obesity exhibited similar key metabolic parameters, including HOMA-IR and insulin levels. This underscores the need to refine the metabolic assessment of pregnant women, moving beyond p-BMI as the primary criterion. A more individualized approach could enhance early risk identification and guide tailored interventions. Our results further support the concept of metabolic subtypes in pregnancy, which may require distinct clinical management strategies, similar to those currently being proposed for non-pregnant adults [6,29]. First-trimester TG and HDL-chol concentrations were within normal ranges and did not differ from those of normal-weight women, though this finding may be biased considering the small sample of women in the GDM group.

An obvious limitation of our study is its design, considering that GDM was one of the metabolic outcomes (GDM) used for selecting study groups. In addition, the strict criteria to be included in the GDM group (having obesity and insulin resistance in the first trimester and developing GDM) resulted in a small sample size, with only 9 out of 108 women meeting these criteria, even though all cases from the cohort with these characteristics at the time of the analysis were included. The lack of association between first-trimester weight and metabolic markers with the risk of GDM may be related to the few numbers with the outcome. The design did not allow us to study all women in the cohort, limiting the generalizability of our findings. A prospective design including women with diverse metabolic risk profiles early in pregnancy is needed to better evaluate FFA trajectories and their association with GDM risk. In addition, we did not assess individual FFA, which may have introduced more variability in the results, potentially masking important associations. Another limitation was that we did not consider diet or physical activity, which both influence lipid and glucose metabolism.

The nesting within a prospective cohort can also be considered a strength, as it allowed us to characterize FFA levels by examining the maternal metabolic risk profiles in the first trimester, while also including women who developed GDM. The clear associations observed between metabolic groups and FFA trajectories highlight the importance of a better metabolic assessment in the beginning of pregnancy, besides BMI, to identify women at higher risk of GDM and other adverse outcomes. Women with obesity and insulin resistance, that developed GDM, exhibited the highest first-trimester FFA levels and the most pronounced decline throughout pregnancy, which indicates metabolic dysfunction since the beginning of pregnancy.

A comprehensive evaluation of metabolic status at the onset of pregnancy, combined with the quantification of FFAs, may enhance the identification of women with obesity at risk of developing gestational diabetes mellitus. This approach could lead to a more precise classification of metabolic risk in the pregnant population, with potential implications for implementing timely and adequate intervention strategies and for initiating close monitoring to reduce the risk of maternal–fetal–infant complications. Further longitudinal studies are needed to confirm these patterns, and to study the behavior of different types of FFAs and their relationship with maternal outcomes and with offspring adiposity.

## 5. Conclusions

FFA concentrations were higher in pregnant women classified with obesity and insulin resistance in the first trimester, that later developed GDM. Longitudinal FFA levels decreased from the first to the third trimester of pregnancy, with the most pronounced decrease observed in women who started pregnancy with obesity and insulin resistance, regardless of developing GDM.

## Figures and Tables

**Table 1:** First-trimester weight and metabolic status of women according to the study group.

	Group 1 (Normal Weight No GDM)	Group 2 (Obesity, No GDM)	Group 3 (Obesity and IR, No GDM)	Group 4 (Obesity, IR, and GDM)	*p*-Value
Age (years)	29.43 ± 5.6	29.30 ± 4.99	27.16 ± 4.10	31.11 ± 5.44	0.202 ^a^
Pregestational BMI (kg/m^2^)	22.63 ± 1.42	34.90 ± 4.38	34.82 ± 3.82	34.35 ± 4.29	<0.001 ^a^
Gestational weight gain (kg)	0.97 ± 4.17	−2.70 ± 5.17	0.13 ± 3.31	2.06 ± 4.78	0.027 ^a^
Glucose (mg/dL)	71.43 ± 10.67	76.39 ± 11.79	84.65 ± 11.74	86.22 ± 15.74	<0.001 ^b^
Insulin (uU/L)	4.46 ± 2.29	5.26 ± 2.61	16.45 ± 7.51	17.87 ± 4.99	<0.001 ^a^
HOMA-IR	0.78 ± 0.40	0.96 ± 0.44	3.49 ± 1.94	3.89 ± 1.67	<0.001 ^a^
Triglycerides (mg/dL)	115.28 ± 31	141.10 ± 32.70	142.02 ± 41.13	133.61 ± 28.00	0.002 ^b^
HDL-Chol (mg/dL)	58.27 ± 12.66	56.32 ± 13.23	55.15 ± 15.24	62.00 ± 16.45	0.597 ^b^

^a^. K–Wallis test/^b^. One-way ANOVA test. BMI—body mass index, HOMA-IR—homeostatic model assessment for insulin resistance, HDL-Chol—high-density lipoprotein, IR—insulin resistance, GDM—gestational diabetes mellitus.

**Table 2:** FFA concentrations during pregnancy by study group.

	Serum FFA (mmol/L)			
	First Trimester ^a^	Second Trimester ^b^	Third Trimester ^a^	Mean Difference ^c^
Group 1	0.403 ± 0.21 *	0.336 ± 0.13 *	0.291 ± 0.13 *	−0.118 ± 0.21
Group 2	0.519 ± 0.20	0.408 ± 0.15	0.340 ± 0.13	−0.282 ± 0.22
Group 3	0.523 ± 0.16	0.417 ± 0.17	0.365 ± 0.13	−0.157 ± 0.18
Group 4	0.657 ± 0.24 *	0.525 ± 0.18 *	0.427 ± 0.08 *	−0.230 ± 0.21
*p*-value	^a^ 0.002 * 0.005	^b^ 0.016 * 0.014	^a^ 0.013 * 0.029	^b^ 0.090

^a^ One-way ANOVA, * post hoc Bonferroni/^b^ K–Wallis test, * Wilcoxon test. ^c^ Serum FFA concentrations in the third trimester minus the concentrations in the first trimester.

**Table 3:** Logistic regression models evaluating the association between first-trimester maternal metabolic risk markers and the risk of gestational diabetes mellitus.

	Coefficient	SE	*p* Value	95% CI
Model 1	Pseudo R^2^: 0.304			
p-BMI	0.088	0.07	0.261	−0.06, 0.24
HOMA-IR	0.407	0.22	0.066	−0.028, 0.843
FFA	3.996	3.6	0.271	−3.113, 11.106
GWG	0.136	0.09	0.150	−0.049, 0.323
TG	−0.0006	0.008	0.939	−0.016, −0.015
Model 3	Pseudo R^2^: 0.327			
p-BMI	0.143	0.085	0.091	−0.023, 0.311
HOMA-IR	0.416	0.259	0.108	−0.092, 0.925
Change FFA	−3.436	2.049	0.094	−7.452, 0.580
GWG	0.207	0.097	0.033	0.017, 0.399
TG	−0.002	0.009	0.751	−0.020, 0.014

p-BMI—pregestational body mass index. HOMA-IR—homeostatic model assessment for insulin resistance. FFA—free fatty acids. GWG—gestational weight gain. TG—triglycerides.

## Data Availability

The data presented in this study are available on request from the corresponding author. The data are not publicly available due to ethical reasons in accordance with consent provided by participants on the use of confidential data.

## Abbreviations

The following abbreviations are used in this manuscript:

BMI	Body mass index
OBESO	Origen Bioquímico y Epigenético del Sobrepeso y la Obesidad
IADPSG	International Association of Diabetes and Pregnancy Study Groups
FFA	Free Fatty Acids
GDM	Gestational Diabetes Mellitus
HOMA-IR	Homeostatic Model Assessment for Insulin Resistance
LMM	Linear Mixed-Effects Model
TG	Triglycerides
HDL-C	High Density Lipoprotein Cholesterol
IR	Insulin Resistance
GWG	Gestational Weight Gain
pBMI	Pregestational Body Mass Index
OGTT	Oral glucose tolerance test
OR	Odds Ratio
CI	Confidence Interval
SMD	Standardized mean difference
IOM	Institute of Medicine
